# Wildlife Roadkill in Chitwan, Nepal: Identifying Affected Species, Potential Drivers and Hotspots

**DOI:** 10.1002/ece3.73709

**Published:** 2026-06-02

**Authors:** Jagan Nath Adhikari, Suman Khanal, Babita Poudel, Dina Nath Dhakal, Bishnu Prasad Bhattarai, Ravi Mohan Tiwari, Chiranjibi Prasad Pokherael

**Affiliations:** ^1^ Department of Zoology Birendra Multiple Campus, Tribhuvan University Bharatpur Chitwan Nepal; ^2^ Nepal Zoological Society Kathmandu Nepal; ^3^ Central Department of Zoology Tribhuvan University Kathmandu Nepal; ^4^ Himalayan Environment and Public Health Network Chitwan Nepal; ^5^ National Trust for Nature Conservation (NTNC) Lalitpur Nepal

**Keywords:** habitat types, mammals, National Highways, road condition, vehicle wildlife collision

## Abstract

Roadkill poses a significant threat to species conservation. However, there is scarce information on roadkill of wild animals. Therefore, to address these research gaps, this study aimed to assess roadkill status of vertebrates, including their conservation status and possible drivers such as seasons, structure of road and habitat types, using direct observation methods over four seasons (autumn, winter, summer and spring) in 2024–2025 on two national highways: part of East–West Highway (H01) and Madan Ashrit Highway (H05). The status of road‐killed vertebrates was evaluated by using relative frequency and encounter rate. The seasonal difference among the reported road‐killed vertebrates were visualized using venn diagram. The generalized linear model was used to show the relationship between total number of roadkill cases of vertebrates with different variables such as seasons, habitat types and condition of road. Similarly, this study also mapped the roadkill hotspots based on data using the Inverse Weighted Distance (IDW) algorithms. A total of 618 cases involving 36 species of wild vertebrates were reported on two highways, comparatively higher in H01. Among the reported species, 36.1% were mammals following 27.78% Aves, 25% reptiles and 11.1% amphibians. This study also recorded two globally Near Threatened (NT), four nationally Vulnerable (VU), and two nationally Near Threatened (NT) roadkill vertebrates. Roadkill risk is closely associated with habitat types. The road sections that cross between forests, forest edges, and residential areas comparatively have higher roadkill cases than croplands. River crossings are critical risk zones for wildlife vehicle encounters. Major roadkill hotspots on both national highways were identified, and recommendations were made for implementing speed limits and constructing underpasses and overpasses during road renovations.

## Graphical abstract

A total of 618 cases involving 36 species of wild vertebrates were reported on sections of National Highways H01 and H05. Comparatively, species richness in road kills was higher on highway H01 (*n* = 343) than on H05 (*n* = 275). The road sections that cross between forests, forest edges, and residential areas consistently increase the number of collisions. Major roadkill hotspots on both national highways were identified, and recommendations were made to implement speed limits and to construct underpasses and overpasses during road renovations.

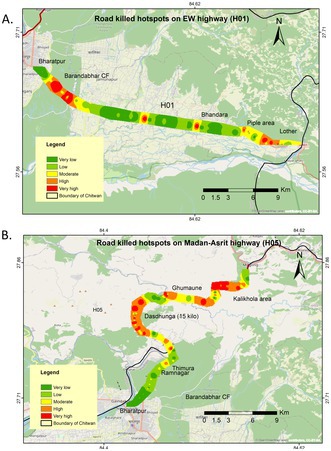

## 1 Introduction

The most visible consequence of roads on wildlife is the vehicle‐induced mortality (Smith and Van Der Ree 2015). Each year, billions of wildlife fatalities are documented globally (Lawton 2018; Oddone Aquino and Nkomo 2021). Roadkill can have a key impact on population dynamics of wildlife and substantially increase the risk of local population decline or even extinction of various threatened species (Fabrizio et al. 2019; Oddone Aquino and Nkomo 2021). In addition, controlling vehicle speed in wild animal crossing areas can help reduce collisions in some taxa, particularly larger vertebrates; however, its effectiveness may be limited for smaller or slow‐moving species such as amphibians (Monge‐Nájera 2018).

Population growth and economic development certainly increase demand for transportation facilities making road construction and expansion (Coale and Hoover 2015; Krukowicz et al. 2022; Mahtta et al. 2022). However, the expansion of roads is considered a sign of economic and social progress, it significantly impacts the landscapes and degrades the natural habitats of threatened species (Monge‐Nájera 2018). Beyond habitat loss, fragmentation and environmental degradation, road construction also affects the mobility and behavior of both wild and domestic animals. The increasing road network leads to increased traffic pressure and increase conservation challenges (da Rosa and Bager 2012).

In developing countries like Nepal, an efficient road transport sector is anticipated to enhance socioeconomic development (Acharya 2021). The main aim of connecting rural areas with road networks is to link agricultural production with markets. Ensuring market access is essential in a country where nearly 78.9% of the total population lives in rural areas and is actively engaged in agriculture‐related industries (Bank 2020). According to the National Highway Statistics for the year 2020/21, Nepal has a network of 80 National Highways with a cumulative length of 14,913 km (SNH 2023). While these highways connect major residential, urban, and suburban areas, they also bisect forests, landscapes, and protected areas thereby increasing the likelihood of wildlife vehicle collisions.

Standardized roadkill data collection at national scales can support meta‐analyses (Forman et al. 2003) on how transport infrastructure affects ecosystem functioning and encourage more ecologically sensitive road development in forested and ecotonal areas (D'Amico et al. 2015). Such evidence can prompt relevant authorities to consider more ecologically sensitive approaches when developing transport infrastructure in forested and ecotonal areas (Saxena et al. 2019). Understanding the spatial and temporal patterns of wildlife road collisions is essential for identifying hotspots, causal factors, and species‐specific vulnerabilities, providing a foundation for targeted mitigation measures. Therefore, road ecology requires stronger international collaboration and expanded research to integrate ecological considerations into road planning and reduce wildlife mortality as transport networks continue to expand.

In Nepal, the East–West Highway (NH01) and Madan Ashrit Highway (Muglin–Narayangarh Highway) (NH05) are the busiest highways, carrying more than 20,000 vehicles daily (SNH 2023). This study focused on a section of these highways, specifically the Lother to Narayangarh section and Madan Ashrit Highway. This study documents roadkill patterns that can help improve road management and reduce wildlife mortality. The previous roadkill research in Nepal has focused either on the single species (i.e., Rusty‐spotted Cat in Suklaphanta National Park, Nepal (Adhikari et al. 2018), Striped Hyaena in central Tarai of Nepal (Adhikari et al. 2019)) or small sections of the highways (i.e., Gondrang–Tikauli section of East–West highway) (Adhikari et al. 2018, 2019). To date, no comprehensive study has been conducted on the highly trafficked Lother to Narayangarh, section of East–West Highway and the Madan Ashrit Highway, leaving the ecological impacts of linear infrastructure in key wildlife crossing areas poorly understood. Addressing this knowledge gap, the present study aims to (i) document the status and seasonal variations of wildlife roadkill, (ii) analyze the threatened status of killed species, (iii) identify wild animal roadkill hotspots and (iv) evaluate the major factors affecting roadkill occurrence along these highways. The results of this study provide baseline data on roadkill patterns of wild vertebrates and serves as a model applicable to other highways experiencing increasing wildlife vehicle collisions. Moreover, this study offers critical insights for road ecologists, conservationists and infrastructure development authorities, including the Department of Roads.

## 2 Materials and Methods

### 2.1 Study Area

**FIGURE 1:**
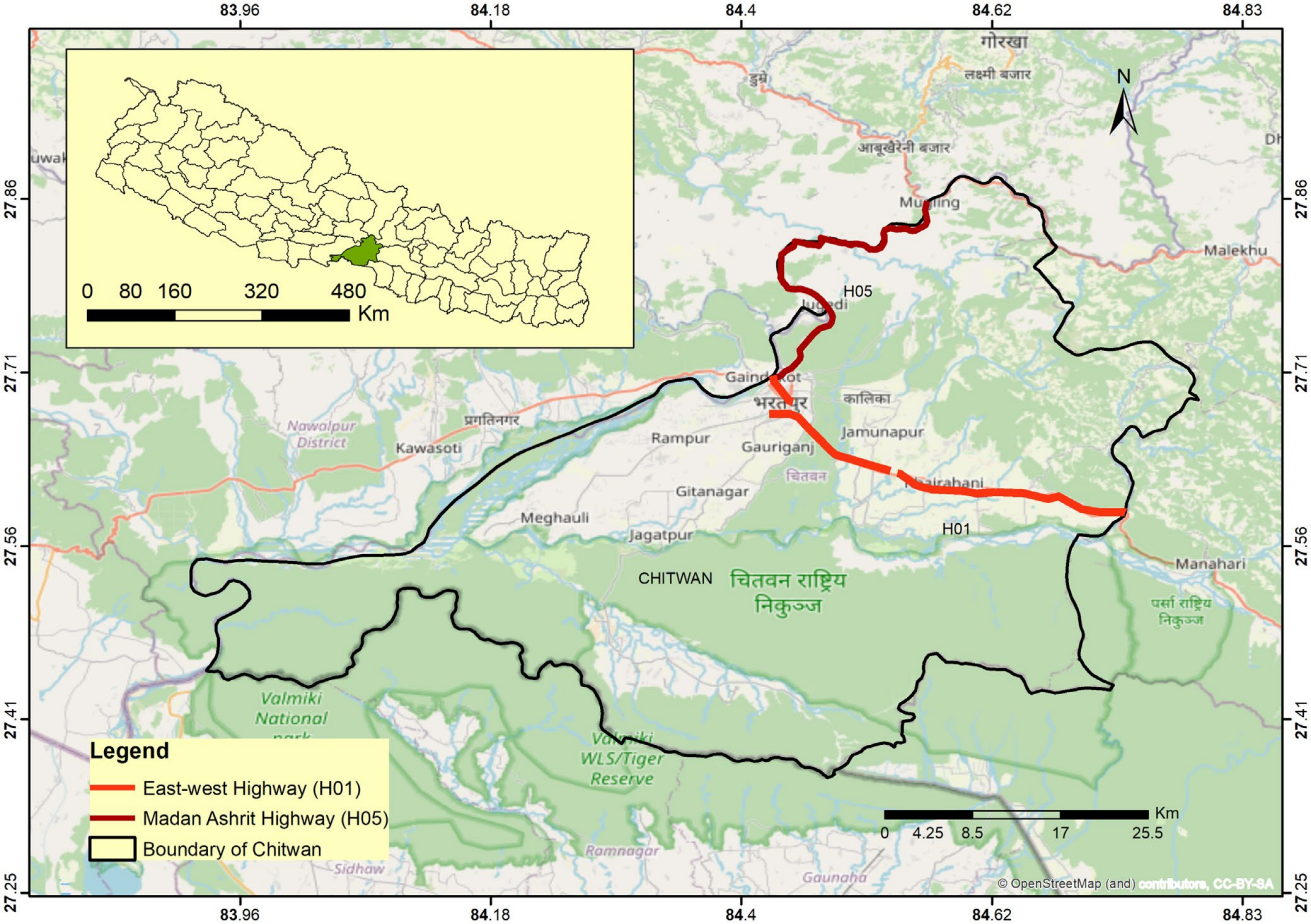
Map showing the Chitwan district along with two national highways H01 and H05.

This study focused on two national highways, East–West Highway (Lother to Narayangarh section) and Madan Ashrit Highway (Figure 1).

The East–West Highway, is also known as the Mahendra Highway, that connects the eastern part—Mechi to the western part—Mahakali of Nepal. It is the longest highway in the country, extending approximately 1030 km in length (SNH 2023). This highway crosses most of the districts of the Tarai along the east–west of Nepal. Lother Narayangarh section is an important section of this highway located in the Chitwan district. It is about 36 km long and crosses the important residential areas such as Rapti Municipality (e.g., Lother, Bhandara), Khaireni Municipality (e.g., Birendra Nagar, Parsa), Ratnanagar Municipality (e.g., Tandi) and Bharatpur Metropolitan City. The highway width in this section ranges from 10 to 30 m. This section bisects the Barandabhar Corridor Forest (BCF), a critical biological corridor connecting Chitwan National Park to the Mahabharat Range. BCF is rich in biodiversity, dominated by Sal forest along with grassland, mixed forest, and wetlands. This area harbors 32 mammal species, 372 bird species and 31 species of herpetofauna (Adhikari et al. 2021; Lamichhane et al. 2016; NTNC 2003; Thapa 2011).

Madan Ashrit Highway is another important national highway (NH05) that connects Narayangarh to Mugling areas and is 36 km long (SNH 2023). Similarly, this road connects two major highways: the East–West Highway and the Prithivi Highway. This section crosses the important residential areas Bharatpur Metropolitan City (e.g., Ramnagar, Jugedi, Bhorle) and Ichhakamana Rural Municipality (e.g., Mugling). Most part of this highway run parallel to the Trishuli River, and crosses the forested landscape that provides habitat for diverse wildlife. Along its route, the highway crosses more than 50 small streams and rivulets and passes through dense forest in particularly in the Ramnagar–Jugedi section. The road generally ranges from 12 to15 m, although it is comparatively wider in urban and semi–urban sections such as Ramnagar, Jugedi.

These two highways (H01 and H05) cross five local units of Chitwan District: Bharatpur Metropolitan city had the highest population (369,368), followed by Ratnanagar Municipality (89,905), Khairahani Municipality (67,385), Rapti Municipality (52,123), and Ichchhakamana Rural Municipality (27,543) (CBS 2021). People in Chitwan District grow food and cash crops such as rice, maize, wheat, lentils, mustard, and vegetables (CBS 2021).

Winter (November to February) is cold in Chitwan, with lower night and morning temperatures and frequent thick fog. The average minimum and maximum temperatures were 8.29°C and 35.48°C, respectively (DHM 2019). Temperature rises until May, remains relatively stable from June to September, and declines thereafter. The monsoon extends from mid‐June to late September, contributing 81.53% of the annual rainfall 1889.23 mm (DHM 2019). The average monthly relative humidity was 76.6%, the lowest in April–May and highest in December–January (DHM 2019). Chitwan is dominated by subtropical vegetation, linking early successional floodplain communities with Sal ( *Shorea robusta* ) – dominated forests, the ecological climax community of Nepal. Other major vegetation types include riverine forest, grassland, and mixed forest. Sal forests are associated with Tatari (*Dillenia pentagyna*), Saaj (*Terminalia alata*), Kyamuna (*Cleistocalyx operculatus*), Karma (*Haldina cordifolia*), Chiraunjee ( *Buchanania latifolia* ), Bhalayo ( *Semecarpus anacardium* ), and Deri ( *Derris elliptica* ). The riverine forest is associated with Vellar (*Trewia nudiflora*), Sisso ( *Dalbergia sissoo* ), Khayer ( *Acacia catechu* ), Simal ( *Bombax ceiba* ), Palas ( *Butea monosperma* ), Pidar (*Xeromphis uliginosa*), Datingal (*Ehretia laevis*), Peepal ( *Ficus religiosa* ), Kutmero (*Listea monopetala*), and Madise‐khirro ( *Holarrhena pubescens* ) (Adhikari 2022).

The surrounding forest around the two national highways supports mammals such as Tiger ( *Panthera tigris* ), Leopard ( *Panthera pardus* ), Sloth Bear (*Ursus ursinus*), Jungle Cat ( *Felis chaus* ), Golden Jackal ( *Canis aureus* ), Large Indian Civet ( *Viverra zibetha* ), Small Indian Civet ( *Viverricula indica* ), Greater One‐horned Rhino ( *Rhinoceros unicornis* ), Wild Boar ( *Sus scrofa* ), Chital ( *Axis axis* ), Sambar ( *Rusa unicolor* ), Northern Red Muntjac (*Muntiacus vaginalis*), Rhesus Macaque ( *Macaca mulatta* ), and Langur (*Semnopithecus* spp). This area supports birds such as Indian Vulture (*Gyps indicus*), White‐rumped Vulture (*Gyps bengalensis*), Yellow‐breasted Bunting ( *Emberiza aureola* ), Greater spotted eagle (*Clanga clanga*), Great Hornbill (*Buceros bicornis*), Lesser Adjutant (*Leptoptilos javanicus*), and Asian Wooly‐necked ( *Ciconia episcopus* ) (Adhikari 2022).

Both highways are two‐lane with a speed limit of 60 km/h along most sections. However, within the Barandabhar corridor forest (approx. 4.8 km), the government of Nepal, in coordination with the Department of National Parks and Wildlife Conservation (DNPWC) and the Department of Forests, has imposed a speed limit of 40 km/h and implemented a no‐horn policy to minimize disturbance and reduce wildlife–vehicle collisions. Some guidelines suggest that horn use may alert certain medium and large‐sized animals; however, animals' response to sound is highly specific to species and may be unpredictable (Jacobson et al. 2016). In some cases, sudden noise can increase collision risk with animals rather than reduce it (Jacobson et al. 2016; Van Der Ree et al. 2015).

### 2.2 Methods

#### 2.2.1 Data Collection

The study was conducted from July 2024 to June 2025. Two national highways (H01 and H05) were selected for systematic roadkill data collection. The entire length of the two highways (approximately 36 km each) was monitored using motorbikes, with two personnel involved: one driver and another observer. The bike was driven at the speed of 20 ± 5 km/h to allow the observer to detect road‐killed wild animals effectively. The survey was conducted twice a week at regular intervals, during early morning (between 06:00 and 09:00 h) and late afternoon (between 14:00 and 17:00 h). A total of 208 survey visits were completed across the two national highways, with 104 visits on each highway. Each highway was monitored twice a week during the study period. Besides systematic surveys, opportunistic data were recorded through the nonsystematic visits conducted in response to external information. These visits were conducted based on the information from media sources, local people, and government officials, and carried out at any time of day on both highways to document roadkill incidents. However, the data collected from these opportunistic observations were not included in the statistical analysis. The study period was divided into four seasons: summer or rainy (June–August), autumn (September–November), winter (December–February), and spring (March–May) to explore the seasonal variation. When roadkill was detected, the bike stopped, and the wild animal's carcass was observed carefully. The dead animals were identified by using the field guide books and identification keys (e.g., Herpetofauna of Nepal by Shah and Tiwari (2004), birds of Nepal by Inskipp et al. (2016) and Grimmett et al. (2016), mammals of Nepal Baral and Shah (2008)). The photographs of each roadkill incident were taken, and the geographic coordinates of the location were recorded using a Global Positioning System (GPS). To avoid multiple counts of the same individuals, the carcasses were removed from the road surface and deposited on the roadside so that the conservation authority can pick them up (Figure S1). The carcasses were not collected in the field, just recorded species, the conditions of the carcass and visible injuries and documented photographically.

The zoological name, order, family along with IUCN and NRDB (National Red Data Book) threatened species were verified using the authentic sources such as http://www.iucnredlist.org/, The Status of Nepal's Mammals: The National Red List Series, https://www.himalayannature.org/citizen‐science/natonal‐red‐list‐of‐birds/ and https://amphibiaweb.org/. Variables such as season, habitat type, and road structure were recorded during the field study (Table 1).

**TABLE 1:** Different types of variables reported during field study.

SN	Variables	Description and types
1	Seasons	Summer or rainy (June–August)
		Autum (September–November)
		Winter (December to February)
		Spring (March–May)
2	Habitat types	Forest: Road crossing along the forest area (i.e., both sides of the road have forest)
		Forest edge: One side has forest and other side has either settlement or cropland
		Cropland: Cultivated area
		Residential area: Settlement area
		River crossing: road that passes through the rivers or streams
3	Road structure	Straight
		S‐mode or sigmoid mode: having bends or turns

#### 2.2.2 Analysis of Data

##### 2.2.2.1 Relative Frequency and Encounter Rate

Relative frequency is the proportion of a species with respect to the total individuals of all species in a specific area (O'Brien 2011). The relative frequency of the roadkill species was calculated as follows:

$$\text{Relative frequency}=\frac{\mathrm{Isi}}{\sum \mathrm{Nsi}}\times 100$$

Here, Isi = Total number of individuals killed by vehicle collision of a species in a total length of a road.

∑ Nsi = Total individuals of all species killed by vehicle collision.

The encounter rate (ER) of roadkill species was calculated by dividing the number of individuals killed in vehicle collisions by the total length of the highway (Fewster et al. 2009).

$$\text{Encounter rate}\;\left(\mathrm{ER}\right)=\frac{n}{L}$$

Here, *n* = total number of road‐killed individuals recorded.

*L* = total length of the road.

##### 2.2.2.2 Generalized Linear Model (GLM)

The generalized linear model (GLM) was used to examine the relationship between the total number of road‐killed vertebrate cases and explanatory variables, including season, habitat type, and road condition. Model coefficients, standard errors and *p*‐values were estimated at a 95% confidence level. Prior to final model selection, the dispersion of the data was assessed and found the data was overdispersed (Range = 107 on H01 and 41 on H05). Therefore, we used a negative binomial error distribution preferred over the Poisson model. The «pscl» package (Jackman 2020) was used for testing overdispersion and evaluating zero‐inflation. The GLM was fitted using the glm() function. All analyses were conducted in R software version 4.2.0 (R Core Team 2025).

##### 2.2.2.3 Venn Diagram

To examine the overlap of road‐killed species among the four seasons, Venn diagrams were generated in R. The venn.diagram() function (Chen and Boutros 2011) was used for general analysis. For improved visualization, the ggVennDiagram (Gao et al. 2021), integrated with ggplot2 (Wickham 2016), was used to produce high‐quality colored diagrams.

##### 2.2.2.4 Road‐Killed Hotspots

Inverse distance weighted (IDW) was used because it is a simple and effective method to show spatial patterns from point data. It works well when the data are reasonably spread out and is useful for showing areas with higher or lower intensity, rather than predicting exact probabilities (Longley et al. 2015). IDW interpolation algorithms in ArcGIS were applied to spatially estimate the distribution of roadkill events along the study highways which were regarded as hotspots (Huang et al. 2011). The interpolated variable was the frequency of roadkill occurrences recorded at georeferenced locations. The input spatial data consisted of GPS coordinates of roadkill points collected during field study. The IDW assigns greater weight to points closer to the prediction location, while those farther away have less influence (Joseph et al. 2010). These areas were interpolated based on known values of the location and predict the value of the unmeasured areas. IDW uses the measured values and predicts the surrounding with separating color composition (Achilleos 2011; Huang et al. 2011). IDW performed a variable search radius of 50 m including the nearest 12 neighboring points. The analysis was conducted in a projected coordinate system UTM Zone 45 N, WGS 84 datum to maximize spatial accuracy. Hotspots were identified from the IDW output raster by classifying areas with the highest predicted density values as high‐risk zones.

## 3 Results

### 3.1 Status of Roadkill Vertebrates

**FIGURE 2:**
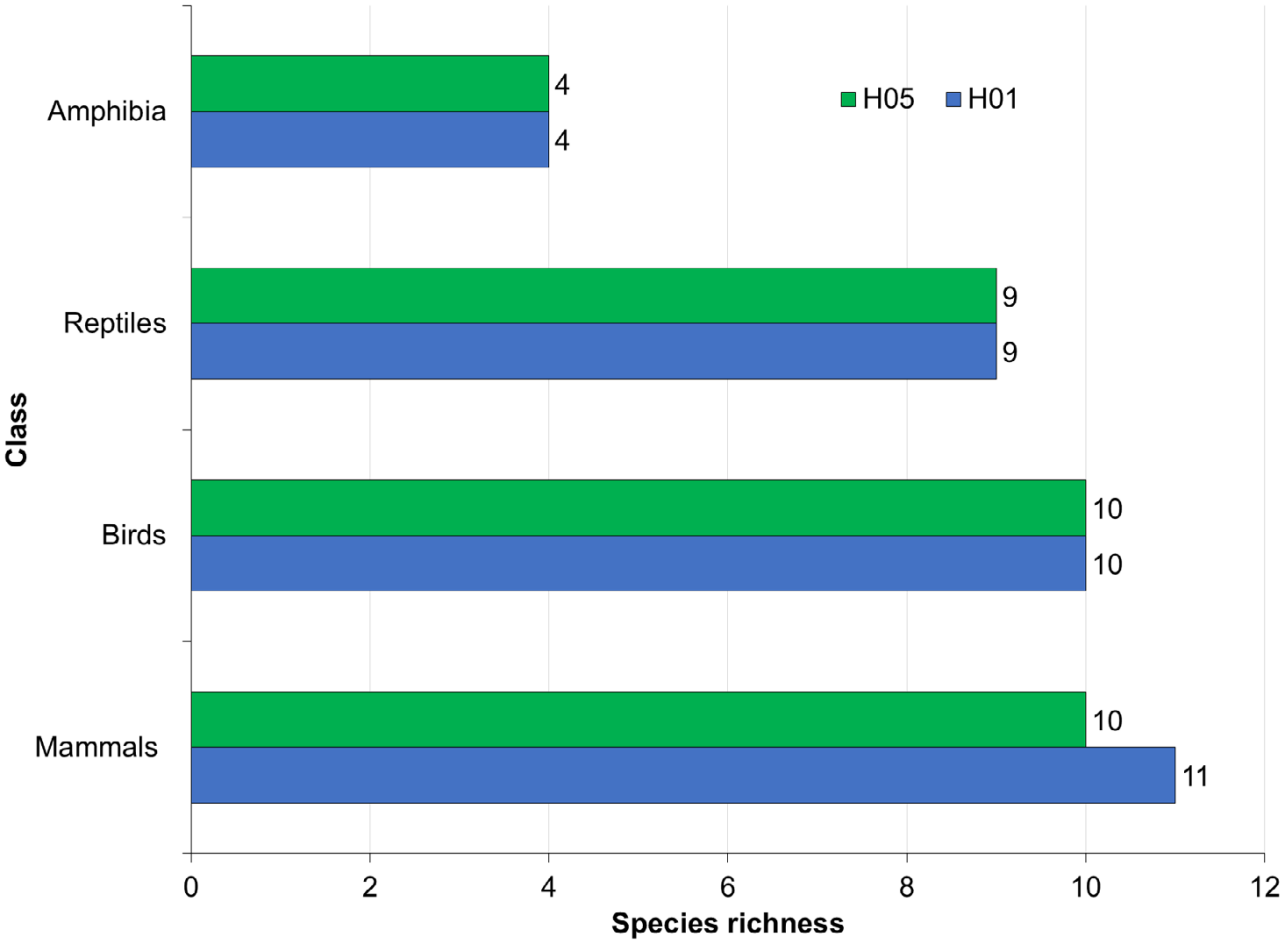
Number of individuals of the vertebrate classes reported in roadkill on two national highways (H01 and H05).

**FIGURE 3:**
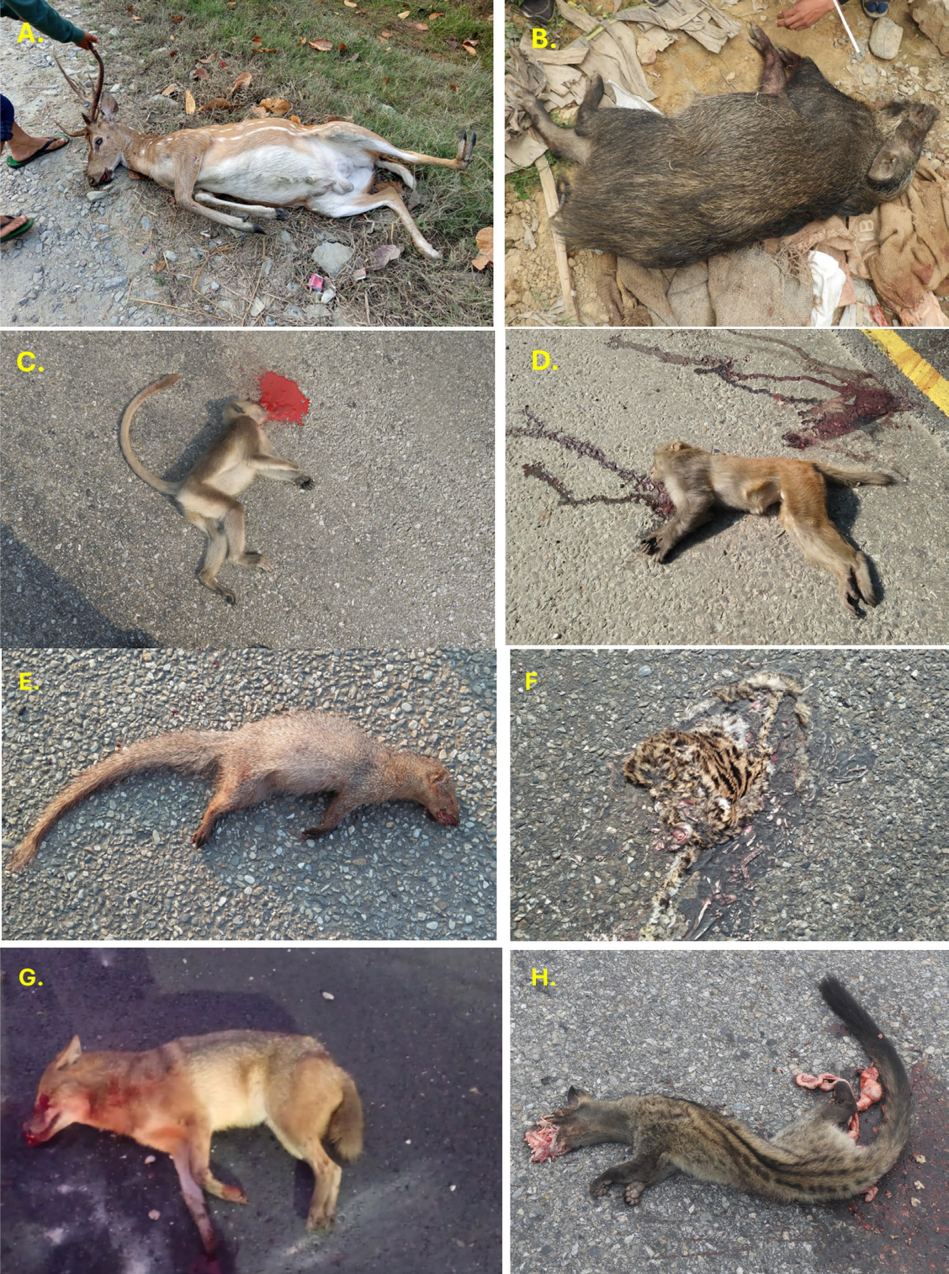
Roadkill Mammals: (A) Chital, (B) Wild boar, (C) Tarai Gray Langur, (D) Rhesus Monkey, (E) Indian Gray Mongoose, (F) Mainland Leopard Cat, (G) Golden Jackal, (H) Small Indian Civet.

**FIGURE 4:**
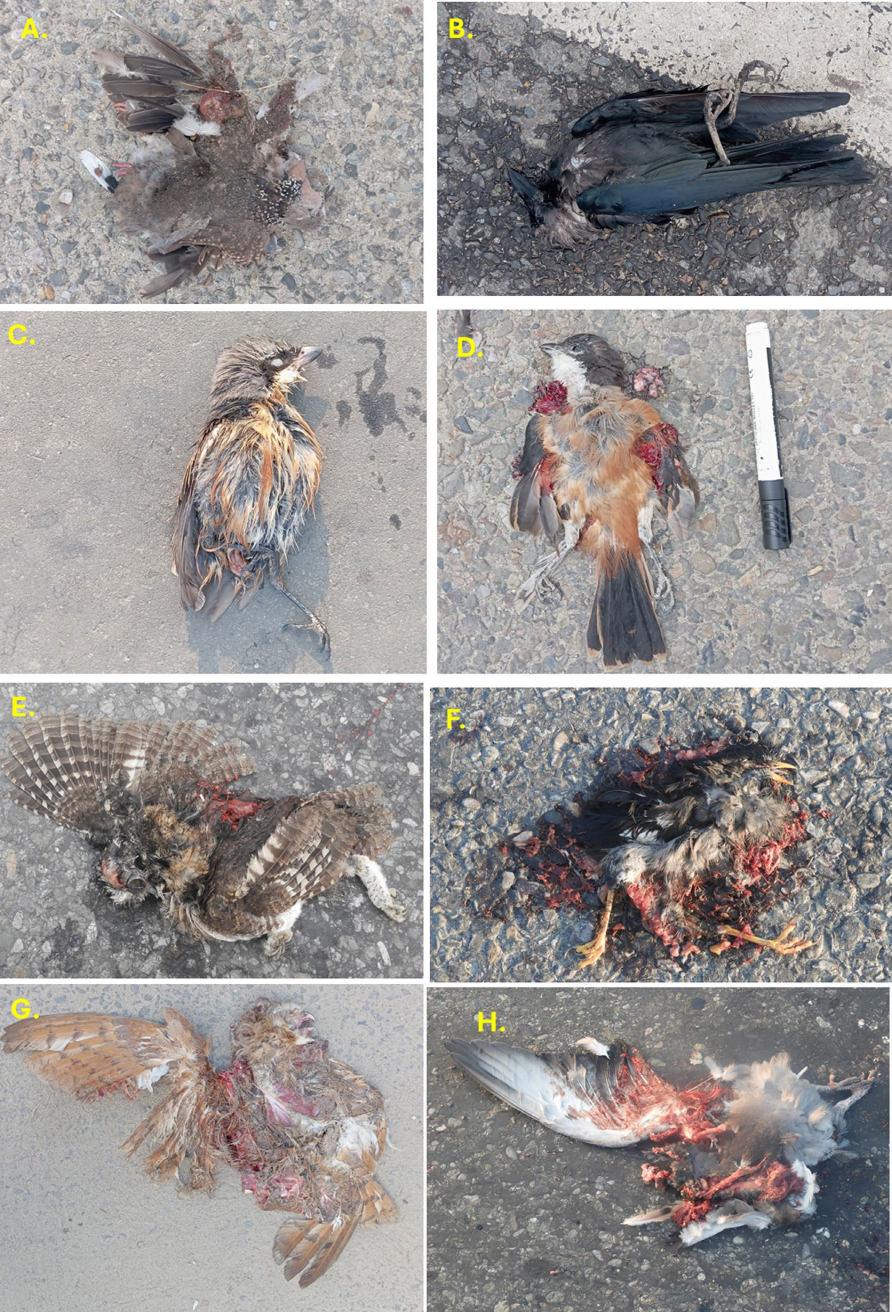
Major birds: (A) Spotted dove, (B) House crow, (C) House sparrow, (D) Long‐tailed shrike, (E) Spotted Owlet, (F) Common Myna, (G) Common Barn‐owl, (H) Rock Dove.

**FIGURE 5:**
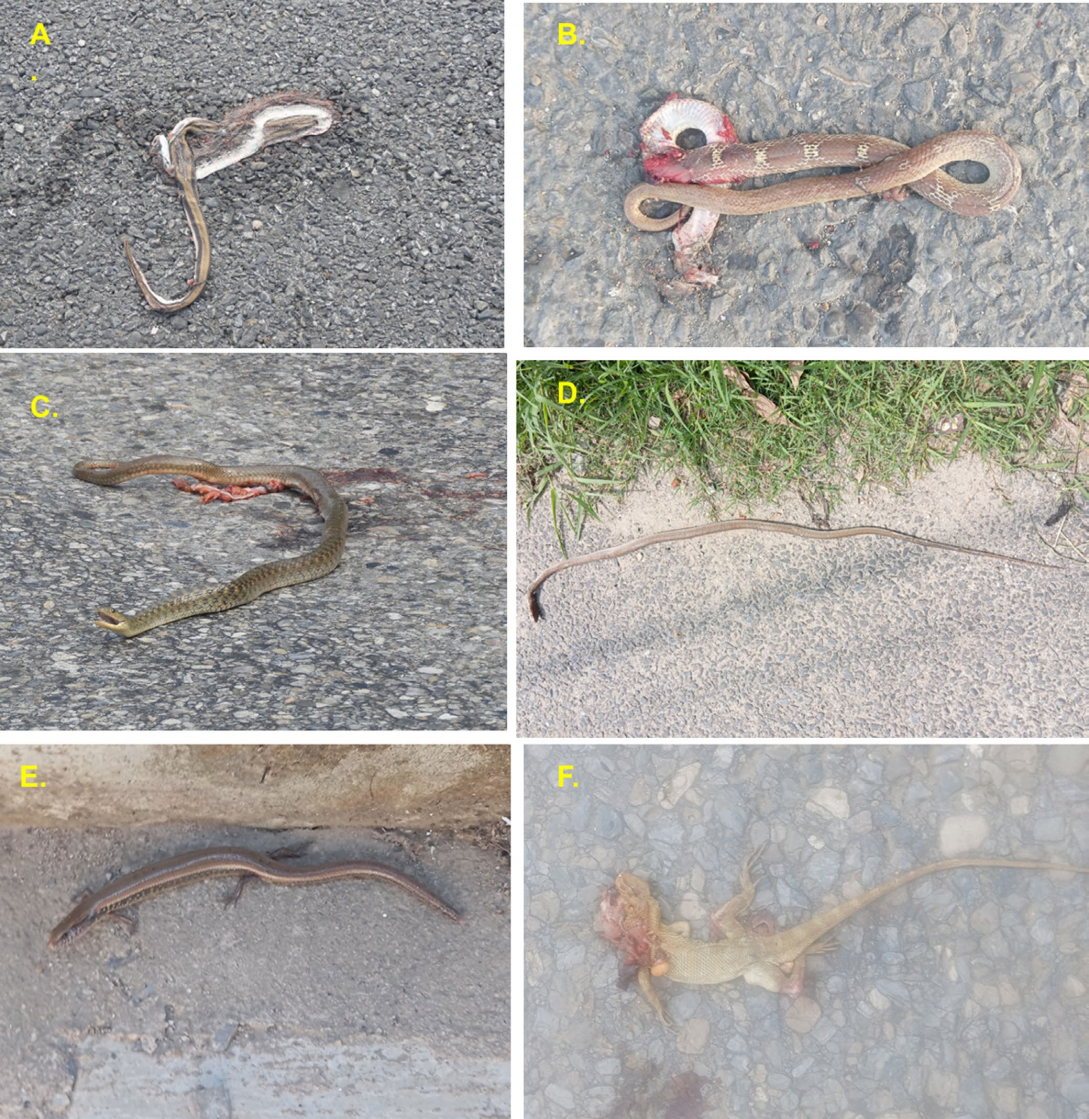
Reptiles killed by vehicle collision: (A) Common trinket snake, (B) Common wolf snake, (C) Checkered keelback, (D) Oriental Ratsnake, (E) Common Snake Skink, (F) Changeable Lizard.

**FIGURE 6:**
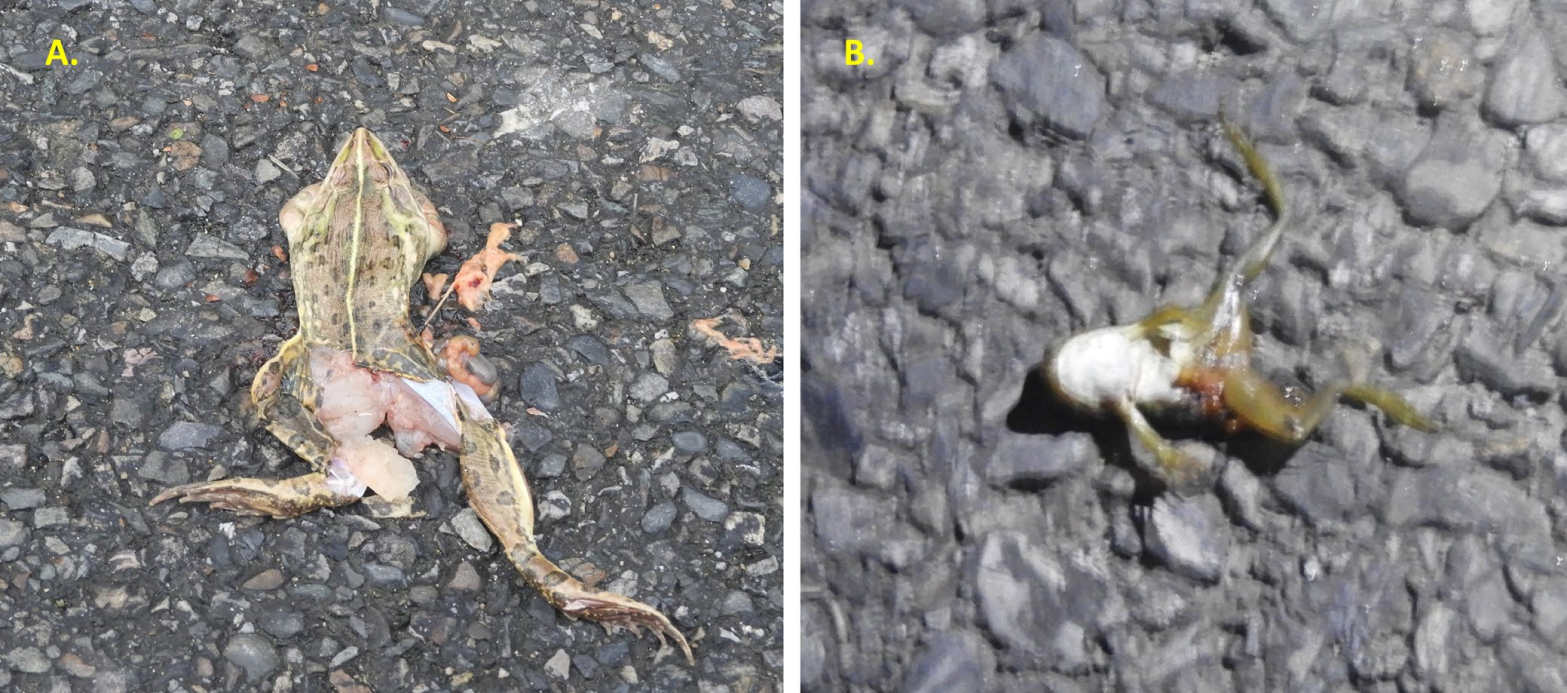
Roadkill: Amphibian, (A) Indian Bullfrog, (B) Skipper Frog.

A total of 618 individuals of 36 species of wild vertebrates were recorded killed during the study period in two sections of National Highway H01 and H05. Comparatively, the number of individuals of the roadkill was higher on highway H01 (*n* = 343) than H05 (*n* = 275). Among the reported 36 types of vertebrates (35 species and one unknown type), 36.1% were mammals, following 27.78% of aves, 25% of reptiles and 11.1% of amphibians (Figures 2, 3, 4, 5). The unidentified species belong to the class Reptiles (e.g., snake) (Table S1). The roadkill vertebrates (*n* = 36 species) belonged to 11 orders, 23 families along with one unknown snake (Table S1). Comparatively, mammals became more victims than aves, reptiles and amphibians (Figures 3, 4, 5, 6).

**FIGURE 7:**
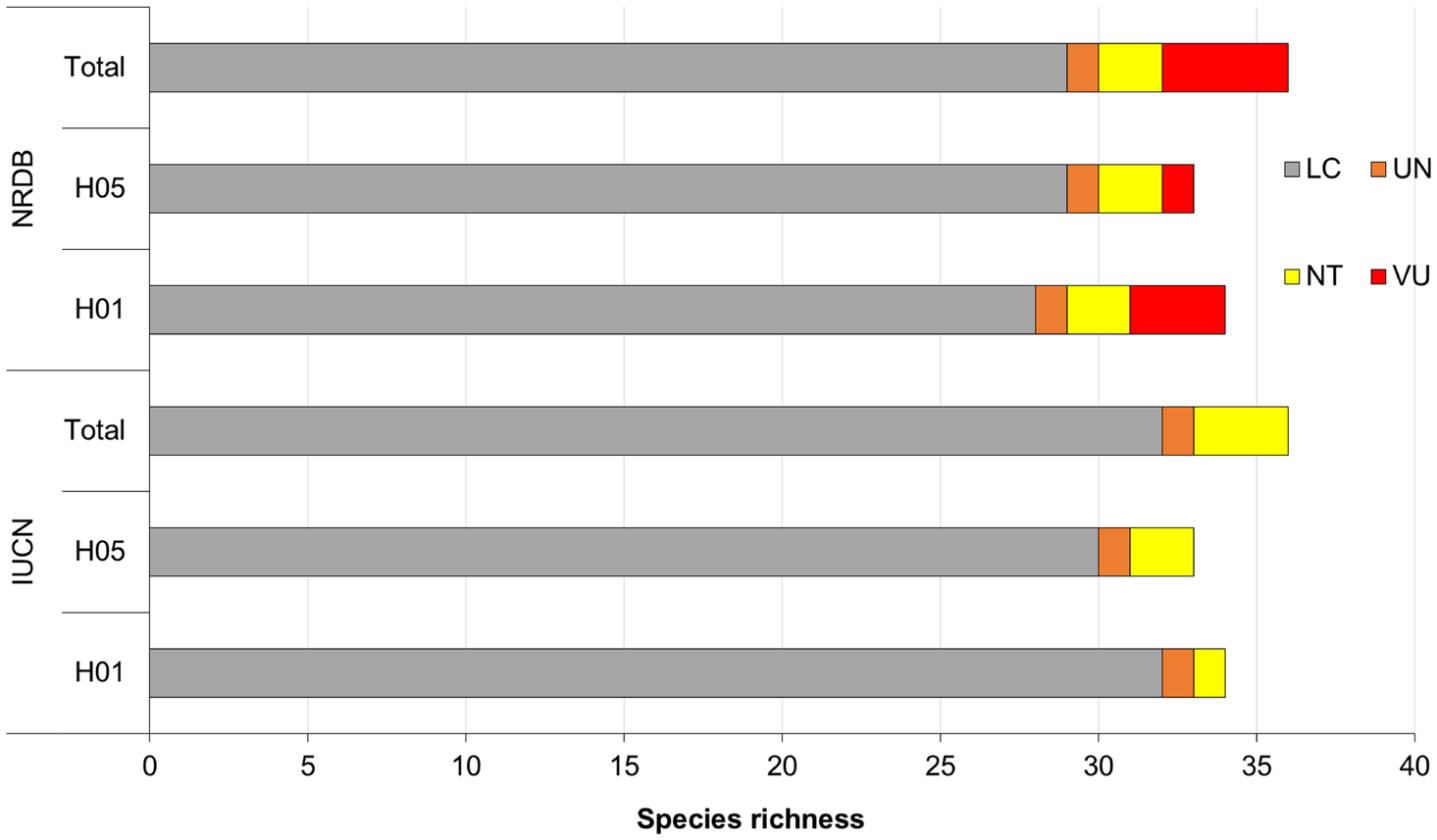
Threatened status of road‐killed vertebrates according to IUCN and NRDB category on two national highways H01 and H05. Here, LC, least concern; NT, near threatened; UN, unknown species; VU, vulnerable.

**FIGURE 8:**
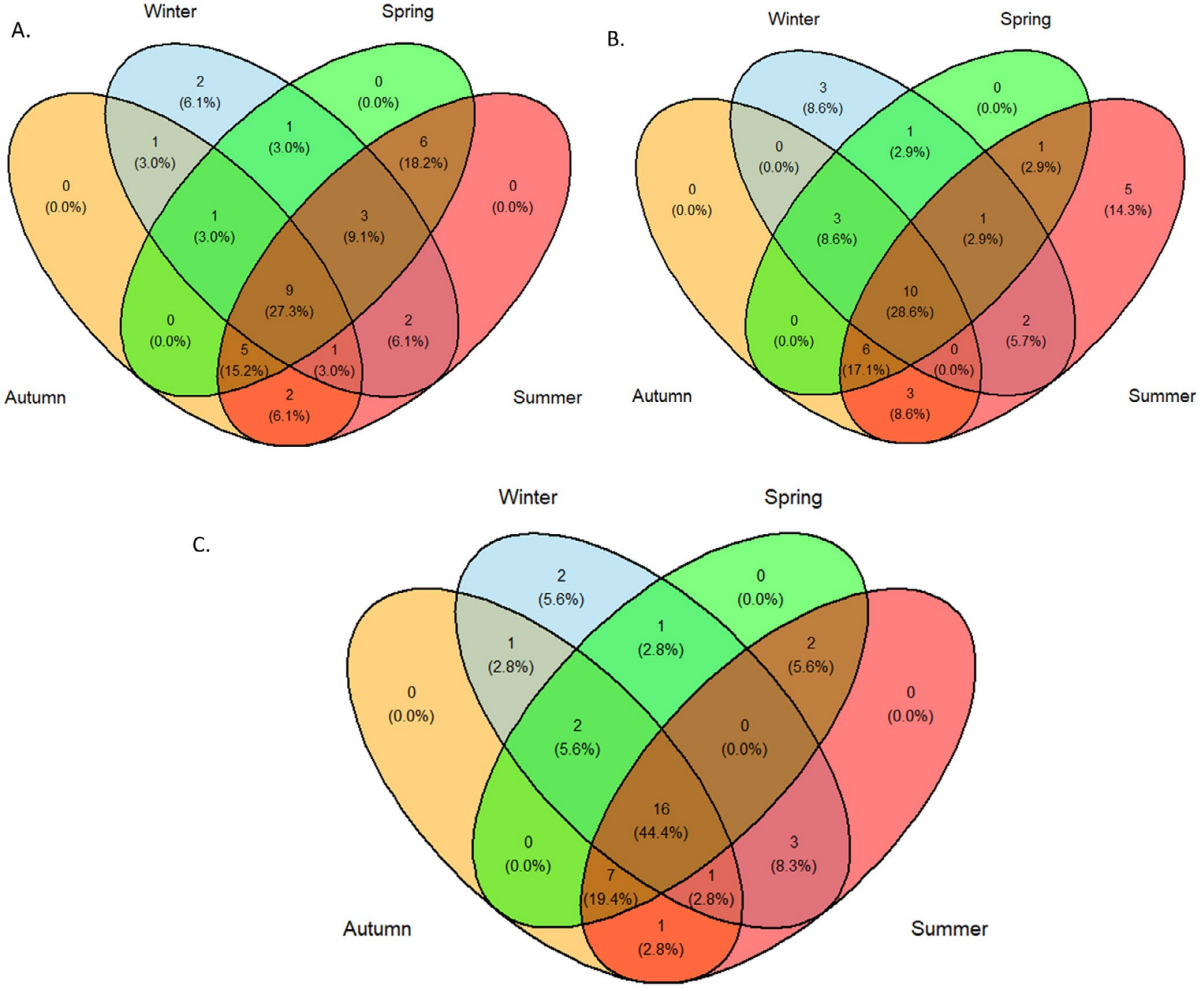
The Venn diagram showing the sharing of road‐killed species between four different seasons—Color‐ coding: Autumn (Orange), Winter (Sky blue), Spring (Green), Summer (Red). (A) Road‐killed vertebrates in H01 (Bharatpur‐Lother section of East–West Highway), (B) Road‐killed vertebrates in H05 (Bharatpur‐Mugling section or Madan‐Ashrit Highway), (C) Total road‐killed vertebrates in both H01 and H05 highways.

**FIGURE 9:**
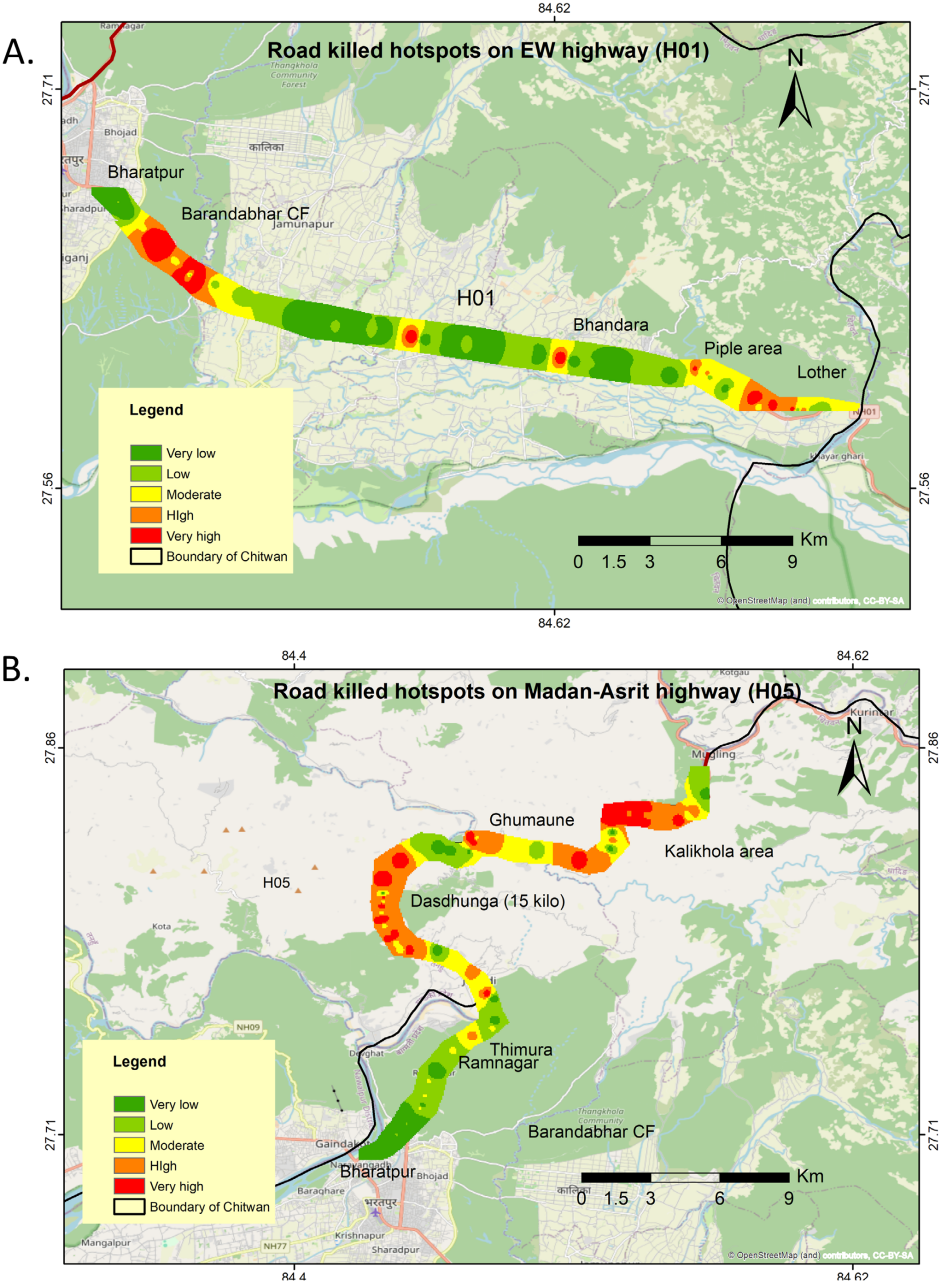
Hotspots of the roadkill developed using IDW. (A) Roadkill hotspot of vertebrate on section of EW highway (H01) and Madan Ashrit highway (H05).

### 3.2 Relative Frequency of Roadkill Vertebrates

Across both H01 and H05 highways, the relative frequency of Indian Bull Frog was the highest, followed by Skipper Frog and Changeable Lizard. Among mammals, the Indian Gray Mongoose was the most frequently recorded road‐killed species. Within avian species, the Rock Dove showed the highest relative frequency of roadkill. On H01, seasonal roadkill patterns varied across taxa. In the autumn season, the Indian Gray Mongoose, Spotted Dove, Common Snake Shink, and Bull Frog showed the highest relative frequencies within mammals, aves, reptiles, and amphibians, respectively. In winter, the Indian Gray Mongoose and Rock Dove dominated mammal and bird roadkill, while only one reptile (Changeable Lizard) and one amphibian (Spotted Tree Frog) were recorded. In spring, Jungle Cat had the highest mammal roadkill, while Rock Dove, Cattle Egret, and Spotted Dove showed equal the highest relative frequencies among birds; Changeable Lizard and Indian Bull Frog dominated reptiles and amphibians. In summer, Golden Jackal, Rock Dove, Changeable Lizard, and Indian Bull Frog showed the highest relative frequencies in their respective groups (Table 2).

**TABLE 2:** Relative frequency (RA, expressed in %), of road‐killed vertebrates on two national highways (East–West Highway, H01 and Madan‐Ashrit Highway, H05) based on seasonal proportion, highway proportion and overall proportion.

SN	Species	Seasonal proportion								Overall proportion		Overall proportion
		Autum		Winter		Spring		Summer				
		H01	H05	H01	H05	H01	H05	H01	H05	H01	H05	
1	Indian Gray Mongoose	3.08	4.55	10	11.11	2.44	3.85	0.53	0.97	2.62	7.55	3.39
2	Jungle Cat	1.54	1.52	6	5.56	4.88	1.92	1.07	3.88	2.33	5.66	2.75
3	Small Indian Civet	1.54	3.03	4	7.41	2.44	3.85	2.14	2.91	2.33	6.91	3.07
4	Large Indian Civet	0	1.52	2	3.7	2.44	1.92	1.07	0.97	1.17	3.14	1.46
5	Wild Boar	0	0	2	0	0	0	0	0	0.29	0	0.16
6	Chital	0	0	0	0	2.44	0	1.07	0	0.87	0	0.49
7	Mainland Leopard Cat	0	0	0	1.85	0	0	0	0	0	0.63	0.16
8	Golden Jackal	1.54	3.03	4	5.56	2.44	3.85	3.21	2.91	2.92	6.29	3.24
9	Tarai Gray Langur	0	1.52	0	5.56	0	3.85	0	3.88	0	6.29	0.16
10	Rhesus Monkey	0	1.52	2	3.7	2.44	5.77	1.07	4.85	1.46	6.29	2.59
11	Brown Spiny Mouse	0	0	2	1.85	0	0	0	0.97	0.29	1.26	0.49
12	Common Palm Civet	0	0	0	1.85	0	1.92	0	0	0	1.26	0.32
13	Bengal Fox	0	0	4	0	0	0	1.07	0	1.17	0	0.65
14	Rock Dove	1.54	0	22	1.85	4.88	0	2.14	3.88	5.25	3.14	3.72
15	House Crow	0	0	2	0	0	0	1.07	0.97	0.87	0.63	0.65
16	Common Myna	3.08	3.03	8	7.41	2.44	5.77	0	0	2.04	5.66	2.58
17	Common Barn‐owl	1.54	0	6	1.85	0	0	0	0	1.17	0.63	0.81
18	Cattle Egret	0	1.52	2	5.56	4.88	1.92	0	0	0.87	3.14	1.29
19	Spotted Dove	4.62	3.03	8	9.26	4.88	1.92	1.07	0.97	3.49	5.66	3.39
20	Red‐vented bulbull	3.08	0	2	3.7	2.44	0	1.07	1.94	1.75	2.52	0.16
21	Long‐tailed Shrike	1.54	0	0	3.7	2.44	5.77	0.53	0.97	0.87	3.77	1.46
22	Spotted Owlet	0	1.52	4	0	2.44	0	1.07	1.94	1.46	1.89	1.29
23	House Sparrow	3.08	0	2	0	0	0	1.07	0.97	1.46	0.63	0.97
24	Snake UN	3.08	7.56	0	1.85	9.76	13.46	2.67	4.85	3.21	11.32	4.69
25	Changeable Lizard	1.54	3.03	6	11.11	14.63	7.69	2.14	0.97	4.08	8.18	4.37
26	Common Snake Skink	3.08	0	0	0	2.44	0	1.07	0.97	1.46	0.63	0.97
27	Oriental Ratsnake	1.54	1.52	0	0	2.44	3.85	3.74	4.85	2.62	5.03	2.75
28	Common Krait	0	0	0	0	2.44	1.92	0.53	1.94	0.58	1.88	0.81
29	Copper‐head Trinket Snake	0	3.03	0	0	2.44	1.92	1.07	5.83	0.87	5.66	1.94
30	Checkered Keelback	0	1.52	0	0	2.44	0	2.14	2.91	1.46	2.52	1.47
31	Trinket Snake	0	1.52	0	0	4.88	1.92	2.67	1.94	2.04	2.52	1.78
32	Common Wolf Snake	0	1.52	0	0	4.88	1.92	1.07	3.88	1.17	3.77	0.16
33	Indian Bullfrog	24.66	25.76	0	0	7.32	3.85	47.59	22.33	31.48	26.42	24.27
34	Asian Common Toad	12.31	4.55	0	0	0	0	2.14	4.85	3.49	5.03	3.24
35	Skipper Frog	21.54	22.73	0	5.56	0	11.54	10.16	6.79	9.62	19.49	10.36
36	Spotted Tree Frog	6.15	1.52	2	0	2.44	9.62	2.67	4.85	3.21	6.29	3.56

In H05, seasonal roadkill patterns also varied among taxa. Gray Mongoose showed the highest relative frequency among mammals in autumn and winter, while Rhesus Monkey dominated in spring and summer. Among birds, Common Myna, Spotted Dove, and Long‐tailed Shrike showed the highest frequencies across seasons. For reptiles, snakes (Unknown) reported the highest relative frequency in autumn and spring, Changeable Lizard in winter, and Copper–head Trinket Snake in summer. Among amphibians, Indian Bull Frog showed the highest relative frequency in all seasons except winter (Table 2).

### 3.3 Encounter Rate of Roadkill Animals

In total, the encounter rate of the road‐killed vertebrates was 8.58/km. Overall, Indian Bull Frog showed the highest encounter rate, particularly on H01 (3/km) and H05 (0.86/km). Among the mammals, Indian Gray Mongoose had the highest encounter rate while Rock Dove (H01) and Common Myna and Spotted Dove on H05 had the highest roadkill among birds. Changeable lizard showed the highest encounter rate among reptiles on both highways. Among the amphibians, Indian Bull Frog had the highest ER on H01 whereas Skipper Frog was the highest on H05 (Table 3).

**TABLE 3:** Encounter rate (ER) of road‐killed vertebrates on two national highways (East–West Highway, H01 and Madan‐Ashrit Highway, H05) based on seasonal proportion, highway proportion.

SN	Species	Seasonal proportion								Highway proportion	
		Autum		Winter		Spring		Summer			
		H01	H05	H01	H05	H01	H05	H01	H05	H01	H05
1	Indian Gray Mongoose	0.06	0.08	0.14	0.17	0.03	0.06	0.03	0.03	0.25	0.33
2	Jungle Cat	0.03	0.03	0.08	0.08	0.03	0.03	0.06	0.11	0.22	0.25
3	Small Indian Civet	0.03	0.06	0.06	0.11	0.03	0.06	0.11	0.08	0.22	0.31
4	Large Indian Civet	0	0.03	0.03	0.06	0.03	0.03	0.06	0.03	0.11	0.14
5	Wild Boar	0	0	0.03	0	0	0	0	0	0.03	0
6	Chital	0	0	0	0	0.03	0	0.06	0	0.08	0
7	Mainland Leopard Cat	0	0	0	0.03	0	0	0	0	0	0.03
8	Golden Jackal	0.03	0.06	0.06	0.08	0.03	0.06	0.17	0.08	0.03	0.03
9	Tarai Gray Langur	0	0.03	0	0.08	0	0.06	0	0.11	0	0.03
10	Rhesus Monkey	0	0.03	0.03	0.06	0.03	0.08	0.08	0.14	0.14	0.31
11	Brown Spiny Mouse	0	0	0.03	0.03	0	0	0	0.03	0.03	0.06
12	Common Palm Civet	0	0	0	0.03	0	0.03	0	0	0	0.06
13	Bengal Fox	0	0	0.06	0	0	0	0.06	0	0.11	0
14	Rock Dove	0.03	0	0.31	0.03	0.06	0	0.11	0.11	0.5	0.14
15	House Crow	0	0	0.03	0	0	0	0.06	0.03	0.08	0.03
16	Common Myna	0.06	0.06	0.11	0.11	0.03	0.08	0	0	0.19	0.25
17	Common Barn‐owl	0.03	0	0.08	0.03	0	0	0	0	0.11	0.03
18	Cattle Egret	0	0.03	0.03	0.08	0.06	0.03	0	0	0.08	0.14
19	Spotted Dove	0.08	0.06	0.11	0.14	0.06	0.03	0.08	0.03	0.33	0.25
20	Red‐vented Bulbull	0.06	0	0.03	0.06	0.03	0	0.06	0.06	0.17	0.11
21	Long‐tailed Shrike	0.03	0	0	0.06	0.03	0.08	0.03	0.03	0.08	0.17
22	Spotted Owlet	0	0.03	0.06	0	0.03	0	0.06	0.06	0.14	0.08
23	House Sparrow	0.06	0	0.03	0	0	0	0.06	0.03	0.14	0.03
24	Snake UN	0.06	0.14	0	0.03	0.11	0.19	0.14	0.14	0.31	0.5
25	Changeable Lizard	0.03	0.06	0.08	0.17	0.17	0.11	0.11	0.03	0.39	0.36
26	Common Snake Skink	0.06	0	0	0	0.03	0	0.06	0.03	0.14	0.03
27	Oriental Ratsnake	0.03	0.03	0	0	0.03	0.06	0.19	0.14	0.25	0.22
28	Common Krait	0	0	0	0	0.03	0.03	0.03	0.06	0.06	0.08
29	Copper‐head Trinket Snake	0	0.06	0	0	0.03	0.03	0.06	0.17	0.08	0.25
30	Checkered Keelback	0	0.03	0	0	0.03	0	0.11	0.08	0.14	0.11
31	Trinket Snake	0	0.03	0	0	0.06	0.03	0.14	0.06	0.19	0.11
32	Common Wolf Snake	0	0.03	0	0	0.06	0.03	0.06	0.11	0.11	0.17
33	Indian Bullfrog	0.44	0.47	0	0	0.06	0.06	2.47	0.64	3	0.17
34	Asian Common Toad	0.22	0.08	0	0	0	0	0.11	0.14	0.33	0.22
35	Skipper Frog	0.39	0.42	0	0.08	0	0.17	0.53	0.19	0.92	0.86
36	Spotted Tree Frog	0.11	0.03	0.03	0	0.03	0.14	0.14	0.14	0.31	0.31

### 3.4 Conservation Status of Kill Animals

Among the reported road‐killed species, two were globally Near threatened (NT) vertebrates—Tarai Gray Langur ( *Semnopithecus hector* ) and Common Krait ( *Bungarus caeruleus* ). Similarly, four nationally vulnerable vertebrates– Mainland Leopard Cat ( *Prionailurus bengalensis* ), Chital ( *Axis axis* ), Bengal Fox ( *Vulpes bengalensis* ) and Common Barn‐owl ( *Tyto alba* ); two nationally near threatened vertebrates– Large Indian Civet ( *Viverra zibetha* ) and Common Krait ( *Bungarus caeruleus* ) were reported during roadkill study (Figure 7). One unidentified reptilian species (e.g., snake) was listed in the unknown (UN) category. Other species were least concern, but the local status is even unknown. Both sections of H01 highway and H05 highway were the death zones for nationally and globally threatened vertebrate species.

### 3.5 Seasonal Variation in Wildlife Roadkill

The GLM results showed significant seasonal effects on roadkill patterns. On H01, autumn, spring, and summer had significant positive effects on roadkill, whereas winter showed no significant effect (Table 4). On H05, spring, summer, and winter were significant predictors, with summer showing the significant positive effects, while autumn was marginally nonsignificant (Table 4). Across both highways, roadkill was the highest in spring and summer, moderate in autumn, and the lowest in winter, indicating clear seasonal variation in roadkill events.

**TABLE 4:** Results of generalized linear model (GLM) showing the effect of different seasons on the number of individuals of different road‐killed vertebrate species (Signif. codes: 0 “***” 0.001 “**” 0.01 “*” 0.05 “·” 0.1 “1”).

Parameters	Estimate	Std. error	*z*	Pr(>|z|)	Significance
**H01**					
(Intercept)	−0.540	0.519	−1.04	0.298	
Autum	0.284	0.068	4.17	< 0.000	***
Spring	1.066	0.458	2.327	0.012	*
Summer	0.465	0.1300	3.574	< 0.000	***
Winter	0.781	0.547	1.427	0.153	
**H05**					
(Intercept)	−0.876	0.524	−1.672	0.095	.
Autum	1.217	0.670	1.816	0.069	.
Spring	1.126	0.463	2.431	0.015	*
Summer	0.555	0.162	3.429	< 0.0001	***
Winter	0.885	0.409	2.162	0.031	*
**Total**					
(Intercept)	0.186	0.466	0.398	0.690	
Autum	0.491	0.293	1.674	0.094	.
Spring	0.424	0.202	2.098	0.035	*
Summer	0.261	0.086	3.022	0.002	**
Winter	0.321	0.208	1.54	0.123	

Seasonal patterns showed clear variation in species richness of road‐killed vertebrates. On H01, the highest number of species was reported in summer, followed by spring, winter, and autumn with nine species occurring in all seasons and only two species in winter (Figure 8A). On H05, species richness was also the highest in summer, followed by autumn and spring, with 10 species recorded across all seasons (Figure 8B). Across both highways, a total of 36 species were recorded, with the highest richness in summer, followed by spring and autumn, and the lowest in winter; 16 species were killed in all seasons, indicating substantial seasonal overlap (Figure 8C).

### 3.6 Influence of Habitat Types on Road Mortality Patterns

Roadkill risk showed strong habitat‐related patterns, with forests, forest edges, residential areas, and river crossings generally increasing collision risk, while cropland showed variable effects (Table 5).

**TABLE 5:** Effects of habitat types (cropland, forest, settlement, forest‐edge, and river‐crossing) on roadkill frequency on two highways (H01 and H05) and overall.

Parameters	Estimate	Std. error	*z*	Pr(>|z|)	Significance
**H01**					
(Intercept)	1.82E‐16	1.10E‐08	0	1	
Cropland	1.00E+00	3.07E‐01	3.259	< 0.001	**
Forest	1.00E+00	2.81E‐01	3.558	< 0.0003	***
Resident area	1.00E+00	3.72E‐01	2.686	0.007	**
Forest edge	1.00E+00	4.05E‐01	2.47	0.013	*
River crossing	1.00E+00	2.51E‐01	3.984	< 0.000	***
**H05**					
(Intercept)	0.763	0.144	5.3	< 0.000	***
Cropland	0.0012	0.026	0.055	0.956	
Forest	0.131	0.0501	2.613	0.008	**
Forest edge	0.226	0.045	4.983	< 0.000	***
Resident area	0.102	0.045	2.241	0.025	*
River crossing	0.0612	0.0259	2.361	0.018	*
**Total**					
(Intercept)	1.628	0.105	15.471	< 0.000	***
Cropland	−0.066	0.009	−6.778	< 0.000	***
Forest	0.062	0.019	3.142	0.001	**
Forest edge	0.106	0.018	5.788	< 0.000	***
Resident area	0.141	0.023	6.025	< 0.000	***
River crossing	0.0107	0.009	1.155	0.248	

On H01, all habitat types had a significant positive association with roadkill, with river‐crossing areas and forests showing highly significant effects. On H05, cropland had no significant effect, whereas forest, forest edge, residential areas, and river crossings showed significant effects, with forest edge emerging as the strongest predictor. Across both highways, cropland showed a significant negative association with roadkill, while forest, forest edge, and residential areas remained hotspots. Although river crossings were significant on individual highways, they were not significant in the combined model (Table 5).

### 3.7 Road Structure

Road structure significantly influenced wildlife roadkill abundance, with both straight and sigmoid road segments showing positive effects on mortality. On both H01 and H05, straight and sigmoid roads were highly significant predictors of roadkill, while the combined analysis also showed a strong overall effect on road structure. Although both road types increased roadkill abundance, sigmoid segments generally showed a stronger effect than straight roads (Table 6).

**TABLE 6:** The effects of road structure (straight and sigmoid mode) on the abundance of roadkill on the H01, H05, and in total.

Parameters	Estimate	Std. error	*z*	Pr(>|z|)	Significance
**H01**					
(Intercept)	0.979	0.121	8.144	< 0.000	***
S.mode	0.171	0.034	5.05	< 0.000	***
Straight	0.069	0.018	3.708	0.0002	***
**H05**					
(Intercept)	4.47E‐13	3.33E‐06	0	1	
S.mode	0.989	0.207	4.784	< 0.000	***
Straight	1.03	0.222	4.634	< 0.000	***
**Total**					
(Intercept)	1.484	0.106	14.039	< 0.000	***
S.mode	0.117	0.015	7.596	< 0.000	***
Straight	0.034	0.007	5.185	< 0.000	***

### 3.8 Roadkill Hotspots

The IDW analysis identified roadkill hotspots along both highways (H01 and H05). On East–West Highway (H01), major hotspots were in the Barandabhar Corridor Forest area, Kayer Khola, Bhandara, Piple, and Lother area. Similarly, H05 showed more roadkill hotspots than H01, with major hotspots concentrated in Thimura, Dashdhunga, 15 Kilo, Ghumaune, Jalbire, and Kalikhola (Figure 9).

## 4 Discussion

### 4.1 Status of Roadkill of Vertebrates

This study indicates a high risk of vertebrate mortality along the specific sections of the two national highways highlighting the growing conservation challenge posed by wildlife vehicle collisions in Nepal. Across the four seasons (July 2024 to June 2025), a total of 618 individuals of roadkill vertebrates, including amphibians, reptiles, aves and mammals were recorded. Mammal mortality was higher on H01, likely because this highway passes through densely populated areas, croplands, forests and forest edges including ecologically important areas such as Barandabhar Corridor Forest and adjoining ecotones at Piple, Lother and Bhandara. These heterogenous landscape increase wildlife movement and consequently the risk of collision. In contrast, H05 passes through less populated areas with more continuous forest cover and steep terrain that may limit animal crossings, resulting in comparatively lower mammal mortality. Roads are known to alter species diversity and distribution by fragmenting habitats and displacing sensitive species from their natural habitats (Bartonička et al. 2018). In Nepal, roadkill incidents have been increasing, especially where highways intersect protected forests, corridor forests and protected areas (Adhikari et al. 2019; Magar et al. 2022). The East–West Highway section passing through the BCF, a biologically significant corridor, supports key wildlife species such as ungulates, rhinoceros and tigers (Adhikari et al. 2021). Similarly, Highway, H05 crosses sections of the BCF (Ramnagar and Jugedi) and the Chitwan Annapurna Landscape, an important vertical corridor facilitating wildlife movement. The construction of wildlife underpasses in the Ramnagar and Jugedi areas has also contributed to reducing road mortality in these sections (Thapa et al. 2021).

### 4.2 Seasonal Variation in Roadkill of Vertebrates

Seasonal variation strongly influences animal movement patterns and consequently road mortality (Fryxell and Sinclair 1988). During the winter, grasslands and forests become dry, potentially increasing mammal mortality as individuals expand their range in search for food (Fryxell and Sinclair 1988). Consistent with this, our results showed higher road mortality of mammals during winter on both highways, likely due to increased road crossings associated with foraging behavior. In contrast, amphibian road mortality was the highest during summer and autumn corresponding to their peak activity and breeding period, while mortality was the lowest in winter when amphibians are relatively inactive. Similar seasonal patterns have been reported in previous studies by Eberhardt et al. (2013), Magar et al. (2022), Puky (2005), and Elzanowski et al. (2009). Overall, this study reported 343 individuals of vertebrates killed on H01 and 275 on H05 due to vehicle collisions, with mortality generally higher in summer than in other seasons. Higher amphibian mortality during summer also contributed substantially to the overall roadkill during this season, whereas mammal mortality was higher during winter. Reduced visibility due to thick fog in winter may further increase bird vulnerability, as birds often use open road surfaces for foraging and are thus more prone to collisions (Selvan et al. 2012). Similarly, reptile road mortality was higher during the summer on both highways, likely reflecting greater seasonal activity in warmer periods (Leynaud et al. 2008). Similar results have been reported from Southern India, including studies by Baskaran and Boominathan (2010) and Selvan et al. (2012) in Karnataka, India.

### 4.3 Factors Affecting Wildlife Roadkill

Road structure and surrounding habitat types significantly influence the frequency of wildlife roadkill (Bueno et al. 2015; Orlowski and Nowak 2006). This study found that both straight and sigmoid road sections contribute to higher mortality, although sigmoid segments show a stronger association with roadkill incidence. This effect is more noticeable in H05 than in H01 as the sigmoid road sections are common in H05. Site‐specific factors, including habitat type, traffic intensity, and species distribution, may also influence wildlife mortality patterns. Additionally, sharp bends further increase the risk of collisions (Zimmermann Teixeira et al. 2017).

This study also examined the relationship between roadkill occurrence and surrounding habitat types. Forest and forest edge habitats showed higher roadkill frequencies for mammals and aves, reflecting their role as natural corridors and foraging grounds (Vorko‐Jović et al. 2006).

Residential areas were also associated with increased roadkill, suggesting that species adapted to human‐modified landscapes often encounter roads (Mohanty and Gupta 2015; Vorko‐Jović et al. 2006). In contrast, croplands showed weaker associations with most animal groups except amphibians, likely due to lower species diversity and reduced movement activity of many wildlife species (Brockie et al. 2009). The open areas (i.e., croplands) are less used by many wild species, though they may still support amphibians under suitable conditions (Brockie et al. 2009; Bueno et al. 2015). Amphibian roadkill were higher in H01 than H05, possibly due to the greater extend of croplands (Paddy field) along H01. River‐crossing sites showed distinct patterns, particularly for semi‐aquatic and highly mobile species, highlighting the role of water sources as movement corridors. Overall, the influence of habitat effects on the roadkill pattern appears to be largely site specific rather than uniform across both highways.

### 4.4 Roadkill Hotspots

The main objective of this study was to identify the roadkill hotspots using frequency and density data. Spatial analysis revealed highlighted hotspot areas along sections of H01 and H05 highways in Chitwan District. An Inverse Distance Weighted (IDW) interpolation method was applied to estimate spatial patterns of roadkill density. This widely used approach is effective for predicting values in unsampled areas and identifying spatial hotspots (Li 2021; Zimmermann Teixeira et al. 2017). This algorithm has also been used in studies on human wildlife conflict (Adhikari et al. 2024; Nad et al. 2022) and roadkill hotspot identification (Medinas et al. 2021). In Nepal, this is the first study to apply IDW for roadkill hotspot identification, providing a useful methodological framework model for future research across other highways. The density of mortality hotspots was comparatively higher along H05 than H01, as H05 passes through less populated areas with lower traffic pressure (SNH 2023). However, the total number of roadkill incidents was higher on H01, where incidents were concentrated within specific hotspots. Habitat type and wildlife presence are key drivers of road mortality (Zimmermann Teixeira et al. 2017). Road sections passing through the forest and ecotone areas showed higher mortality rates due to greater wildlife abundance and frequent crossing behaviors (Litvaitis and Tash 2008; Zimmermann Teixeira et al. 2017). In contrast, roadkill incidence was very low in forested areas near Ramnagar and Jugedi, probably due to the presence of wildlife underpasses, which facilitate safer animal crossing (Thapa et al. 2021). Overall, the Barandabhar Corridor Forest (BCF) has emerged as a major mortality hotspot for vertebrates, as consistent with findings also reported by Magar et al. (2022). This study has some limitations: First, it was conducted only along the selected sections of national highways and may not fully represent roadkill patterns across the East–West Highway and other road networks. Secondly, damaged carcasses may have hindered accurate species identification, and genetic analysis to confirm species identity was not undertaken. Third, the use of camera trapping, which could have provided important insights into the behavior and vulnerability of roadkill, was beyond the scope of this study.

## 5 Conclusions

The present study revealed that the mortality of 36 vertebrate species, including globally and nationally threatened species, across all four seasons, highlighting the significant impact of highways on wildlife. These findings clearly indicate that both roads are unsafe for animal movement and contribute substantially to biodiversity loss. The identification of key roadkill hotspots along both highways provides critical insights that can guide the design and implementation of eco‐friendly and wildlife‐friendly road infrastructure. Based on these findings, the study recommends the following recommendations.

- For management authority
  1. Implement simple yet effective migration measures like construction of speed breakers and installation of warning signposts at the identified hotspots.
  2. Integrate wildlife friendly structures including well‐designed effective underpasses or overpasses particularly in the hotspot areas during road upgrading or renovation.
- For public: Drivers are strongly encouraged to adhere to traffic rules in designated wildlife areas and regulations in animal crossing zones and to obey the posted information including speed limits and no horn zones to minimize wildlife vehicle collisions.
- For researchers and students: There remains a significant gap in roadkill data across many forested areas intersected highways. This study recommends the need for similar research in other parts of the country. It can serve as baseline information for future researchers and planning efforts. Continue and systematic monitoring of roadkill along these highways is therefore highly recommended.

## Author Contributions

**Jagan Nath Adhikari:** conceptualization (lead), data curation (equal), formal analysis (lead), funding acquisition (lead), investigation (lead), methodology (equal), project administration (lead), resources (equal), software (equal), visualization (lead), writing – original draft (lead), writing – review and editing (equal). **Suman Khanal:** conceptualization (equal), data curation (equal), methodology (equal), project administration (equal), writing – review and editing (equal). **Babita Poudel:** conceptualization (equal), data curation (equal), investigation (equal), methodology (equal), writing – review and editing (equal). **Dina Nath Dhakal:** data curation (equal), investigation (equal), writing – review and editing (equal). **Bishnu Prasad Bhattarai:** data curation (equal), formal analysis (equal), methodology (equal), writing – review and editing (equal). **Ravi Mohan Tiwari:** data curation (equal), writing – review and editing (equal). **Chiranjibi Prasad Pokherael:** methodology (equal), supervision (equal), validation (equal), writing – review and editing (equal).

## Funding

This work was supported by University Grants Commission‐ Nepal, SRDIG‐80/81‐S&T‐14. Research Management Cell, Birendra Multiple Campus and Help Nepal Association Japan (HNA‐Japan).

## Conflicts of Interest

The authors declare no conflicts of interest.

## Supporting information


**Figure S1:** Fieldwork activities. (A) Removing a carcass to avoid double‐counting. (B) Examining a carcass for species identification and injury assessment. C. A research assistant is photographing a recorded carcass.

**Table S1:** Checklist of vertebrate species recorded as roadkill, including conservation status from the IUCN Red List and the Nepal Red Data Book (NRDB). Here: LC, least concern; NT, near threatened; VU, vulnerable.

## Data Availability

The data is available in DOI: 10.5061/dryad.51c59zwpq.
