# Supplementary figures and images for: Wildlife Roadkill in Chitwan, Nepal: Identifying Affected Species, Potential Drivers and Hotspots
Source: Ecol Evol. 2026 Jun 2;16(6):e73709. doi: 10.1002/ece3.73709 (PMC13239242; doi:10.1002/ece3.73709)

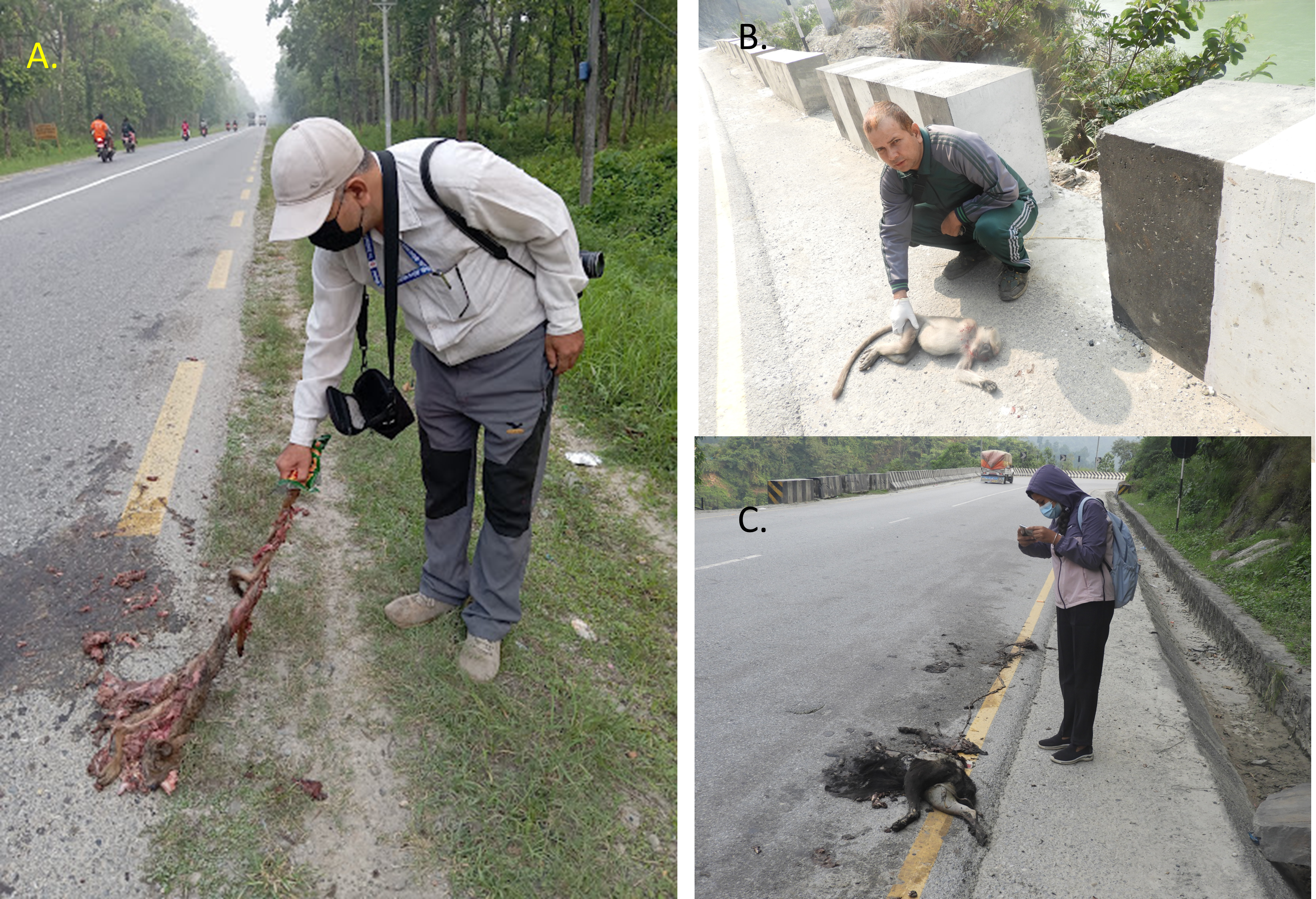

Supplement: Supplementary file 1 — Figure S1: Fieldwork activities. (A) Removing a carcass to avoid double‐counting. (B) Examining a carcass for species identification and injury assessment. C. A research assistant is photographing a recorded carcass. [file ECE3-16-e73709-s002.tif]
