# Supplementary material for: Wildlife Roadkill in Chitwan, Nepal: Identifying Affected Species, Potential Drivers and Hotspots
Source: Ecol Evol. 2026 Jun 2;16(6):e73709. doi: 10.1002/ece3.73709 (PMC13239242; doi:10.1002/ece3.73709)
Supplement: Supplementary file 2 — Table S1: Checklist of vertebrate species recorded as roadkill, including conservation status from the IUCN Red List and the Nepal Red Data Book (NRDB). Here: LC, least concern; NT, near threatened; VU, vulnerable. [file ECE3-16-e73709-s001.docx]

Table S1. Checklist of the vertebrates reported during roadkill

| SN | Order | Family | Common Name | Local name | Zoological name | IUCN | NRDB |
| --- | --- | --- | --- | --- | --- | --- | --- |
| **Mammals** | | | | | | | |
| 1 | Carnivora | Herpestidae | Indian Grey Mongoose | Khairo nyaurimusa | *Herpestes edwardsii* (É. Geoffroy Saint-Hilaire, 1818) | LC | LC |
| 2 | Carnivora | Felidae | Jungle Cat | Jangali Biralo | *Felis chaus* Schreber, 1777 | LC | LC |
| 3 | Carnivora | Viverridae | Small Indian Civet | Sano Nirbiralo | *Viverricula indica* (É. Geoffroy Saint-Hilaire, 1803) | LC | LC |
| 4 | Carnivora | Viverridae | Large Indian Civet | Thulo Nirbiralo | *Viverra zibetha* Linnaeus, 1758 | LC | NT |
| 5 | Carnivora | Felidae | Mainland Leopard Cat | Chari baagh | *Prionailurus bengalensis* (Kerr, 1792) | LC | VU |
| 6 | Carnivora | Canidae | Golden Jackal | Syaal | *Canis aureus* Linnaeus, 1758 | LC | LC |
| 7 | Carnivora | Viverridae | Common Palm Civet | Tadi Nirbiralo | *Paradoxurus hermaphroditus* (Pallas, 1777) | LC | LC |
| 8 | Carnivora | Canidae | Bengal Fox | Phusro Phyuro | *Vulpes bengalensis* (Shaw, 1800) | LC | VU |
| 9 | Cetartiodactyla | Suidae | Wild Boar | Bandel | *Sus scrofa* Linnaeus, 1758 | LC | LC |
| 10 | Cetartiodactyla | Cervidae | Chital | Jarayo | *Axis axis* (Erxleben, 1777) | LC | VU |
| 11 | Primates | Cercopithecidae | Tarai Gray Langur | Langur | *Semnopithecus hector* (Pocock, 1928) | NT | LC |
| 12 | Primates | Cercopithecidae | Rhesus Monkey | Rato Bandar | *Macaca mulatta* (Zimmermann, 1780) | LC | LC |
| 13 | Rodentia | Muridae | Brown Spiny Mouse | Jungali Musa | *Mus saxicola* Elliot, 1839 | LC | LC |
| **Birds** | | | | | | | |
| 14 | Columbiformes | Columbidae | Rock Dove | Parewa | *Columba livia* Gmelin, 1789 | LC | LC |
| 15 | Columbiformes | Columbidae | Spotted Dove | Kurle Dhukur | *Spilopelia suratensis* (Gmelin, 1789) | LC | LC |
| 16 | Passeriformes | Corvidae | House Crow | Kaag | *Corvus splendens* Vieillot, 1817 | LC | LC |
| 17 | Passeriformes | Sturnidae | Common Myna | Dangre | *Acridotheres tristis* (Linnaeus, 1766) | LC | LC |
| 18 | Passeriformes | Passeriformes | Pycnonotidae | Bulbul | *Pycnonotus cafer* (Linnaeus, 1766) | LC | LC |
| 19 | Passeriformes | Laniidae | Long-tailed Shrike | Bhadrai | *Lanius schach* Linnaeus, 1758 | LC | LC |
| 20 | Passeriformes | Passeridae | House Sparrow | Bhangera | *Passer domesticus* (Linnaeus, 1758) | LC | LC |
| 21 | Pelecaniformes | Ardeidae | Cattle Egret | Bakulla | *Bubulcus ibis* (Linnaeus, 1758) | LC | LC |
| 22 | Strigiformes | Tytonidae | Common Barn-owl | Gothe ullu | *Tyto alba* (Scopoli, 1769) | LC | VU |
| 23 | Strigiformes | Strigidae | Spotted Owlet | Khochangande Latokosero | *Athene brama* (Temminck, 1821) | LC | LC |
| **Reptiles** | | | | | | | |
| 24 | Squamata | Scincidae | Common Snake Skink | Bhanemungro | *Lygosoma punctata* (Gmelin, 1799) | LC | LC |
| 25 | Squamata | Agamidae | Changeable Lizard | Chheparo | *Calotes versicolor* (Daudin, 1802) | LC | LC |
| 26 | Squamata | Colubridae | Oriental Ratsnake | Dhaman | *Ptyas mucosa* (Linnaeus, 1758) | LC | LC |
| 27 | Squamata | Elapidae | Common Krait | Karet Sarpa | *Bungarus caeruleus* (Schneider, 1801) | NT | NT |
| 28 | Squamata | Colubridae | Copper-head Trinket Snake | Tamako Tauke Sarp | *Coelognathus radiatus* (Boie, 1827) | LC | LC |
| 29 | Squamata | Unknown |  |  |  |  |  |
| 30 | Squamata | Natricidae | Checkered Keelback, | Water snake | *Fowlea piscator* (Schneider, 1799) | LC | LC |
| 31 | Squamata | Colubridae | Trinket Snake | Shrinagare Sarpa | *Coelognathus helenae* (Daudin, 1803) | LC | LC |
| 32 | Squamata | Colubridae | Common Wolf Snake | Bwase sarpa | *Lycodon aulicus* (Linnaeus, 1758) | LC | LC |
| **Amphibia** | | | | | | | |
| 33 | Anura | Dicroglossidae | Indian Bullfrog | Sirke Paha | *Hoplobatrachus tigerinus* (Daudin, 1802) | LC | LC |
| 34 | Anura | Bufonidae | Asian Common Toad | Khasre Bhyaguta | *Duttaphrynus melanostictus* (Schneider, 1799) | LC | LC |
| 35 | Anura | Dicroglossidae | Skipper Frog | Bhyaguta | *Euphlyctis cyanophlyctis* (Schneider, 1799) | LC | LC |
| 36 | Anura | Rhacophoridae | Spotted Tree Frog | Rukh Bhyaguta | *Polypedates maculatus* (Gray, 1830) | LC | LC |
